# Lysines K117 and K147 play conserved roles in Ras activation from *Drosophila* to mammals

**DOI:** 10.1093/g3journal/jkad201

**Published:** 2023-09-04

**Authors:** Jiya Singh, Prashath Karunaraj, Max Luf, Cathie M Pfleger

**Affiliations:** Department of Oncological Sciences, The Icahn School of Medicine at Mount Sinai, New York, NY 10029, USA; The Tisch Cancer Institute, The Icahn School of Medicine at Mount Sinai, New York, NY 10029, USA; Department of Oncological Sciences, The Icahn School of Medicine at Mount Sinai, New York, NY 10029, USA; The Tisch Cancer Institute, The Icahn School of Medicine at Mount Sinai, New York, NY 10029, USA; The Graduate School of Biomedical Sciences, The Icahn School of Medicine at Mount Sinai, New York, NY 10029, USA; Department of Oncological Sciences, The Icahn School of Medicine at Mount Sinai, New York, NY 10029, USA; The Tisch Cancer Institute, The Icahn School of Medicine at Mount Sinai, New York, NY 10029, USA; Department of Oncological Sciences, The Icahn School of Medicine at Mount Sinai, New York, NY 10029, USA; The Tisch Cancer Institute, The Icahn School of Medicine at Mount Sinai, New York, NY 10029, USA; The Graduate School of Biomedical Sciences, The Icahn School of Medicine at Mount Sinai, New York, NY 10029, USA

**Keywords:** Ras, HRas, NRas, KRas, ubiquitin, *Drosophila*, lysine, K117, K147

## Abstract

Ras signaling plays an important role in growth, proliferation, and developmental patterning. Maintaining appropriate levels of Ras signaling is important to establish patterning in development and to prevent diseases such as cancer in mature organisms. The Ras protein is represented by Ras85D in *Drosophila* and by HRas, NRas, and KRas in mammals. In the past dozen years, multiple reports have characterized both inhibitory and activating ubiquitination events regulating Ras proteins. Inhibitory Ras ubiquitination mediated by Rabex-5 or Lztr1 is highly conserved between flies and mammals. Activating ubiquitination events at K117 and K147 have been reported in mammalian HRas, NRas, and KRas, but it is unclear if these activating roles of K117 and K147 are conserved in flies. Addressing a potential conserved role for these lysines in *Drosophila* Ras activation requires phenotypes strong enough to assess suppression. Therefore, we utilized oncogenic Ras, Ras^G12V^, which biases Ras to the GTP-loaded active conformation. We created double mutants Ras^G12V,K117R^ and Ras^G12V,K147R^ and triple mutant Ras^G12V,K117R,K147R^ to prevent lysine-specific post-translational modification of K117, K147, or both, respectively. We compared their phenotypes to Ras^G12V^ in the wing to reveal the roles of these lysines. Although Ras^G12V,K147R^ did not show compelling or quantifiable differences from Ras^G12V^, Ras^G12V,K117R^ showed visible and quantifiable suppression compared to Ras^G12V^, and triple mutant Ras^G12V,K117R,K147R^ showed dramatic suppression compared to Ras^G12V^ and increased suppression compared to Ras^G12V,K117R^. These data are consistent with highly conserved roles for K117 and K147 in Ras activation from flies to mammals.

## Introduction

Ras signaling is important in development and disease. The KRas, HRas, and NRas genes in mammals are represented by a single Ras gene in *Drosophila* (referred to in the literature as Ras1, Ras85D, and here referred to as Ras). The E3 ubiquitin ligase Rabex-5 inhibits *Drosophila* Ras and mammalian HRas and NRas by promoting their mono- and di-ubiquitination (Jura *et al.* 2006; Yan *et al.* 2009, 2010; Xu *et al.* 2010; Washington *et al.* 2020). The Cul3-Lztr1 ubiquitin ligase also inhibits Ras by ubiquitination in both flies and mammals (Steklov *et al.* 2018; Bigenzahn *et al.* 2018). Thus, multiple means of inhibitory Ras ubiquitination are highly conserved between *Drosophila* and mammals. In addition to inhibitory ubiquitination, mammalian KRas, HRas, and NRas are all reported to be ubiquitinated at lysines K117 and K147 (Akimov *et al.* 2018; Filipčík *et al.* 2017; Wagner *et al.* 2012; Sasaki *et al.* 2011; Baker *et al.* 2013; Udeshi *et al.* 2013; Yoshino *et al.* 2019; Mertins *et al.* 2013). Ubiquitination events at K117 and K147 increase Ras activation by distinct mechanisms. K117 mono-ubiquitination accelerates nucleotide exchange whereas K147 ubiquitination increases GTP-loading and interaction with downstream effectors (Sasaki *et al.* 2011; Baker *et al.* 2013; Hobbs *et al.* 2013; Filipčík *et al.* 2017; Wagner *et al.* 2012).

We report here that individual arginine substitution in K117 in Ras^G12V^, an activated form of Ras, results in phenotypic suppression of Ras^G12V^ phenotypes. Although individual arginine substitution in K147 in Ras^G12V^ did not produce obvious phenotypic suppression of Ras^G12V^ phenotypes, concurrent mutation of both K117 and K147 in Ras^G12V^ results in even greater suppression than individual mutation in K117. These data are consistent with a conserved role for both of these lysines in Ras activation between flies and mammals.

## Materials and methods

### Reproducibility

The reported work represents reproducible experiments that reflect a minimum of 3 well-controlled, independent trials performed by at least 2 different lab members. Many experiments reported here were repeated in excess of 8 times.

### Statistical analysis

**Fig. 1.**
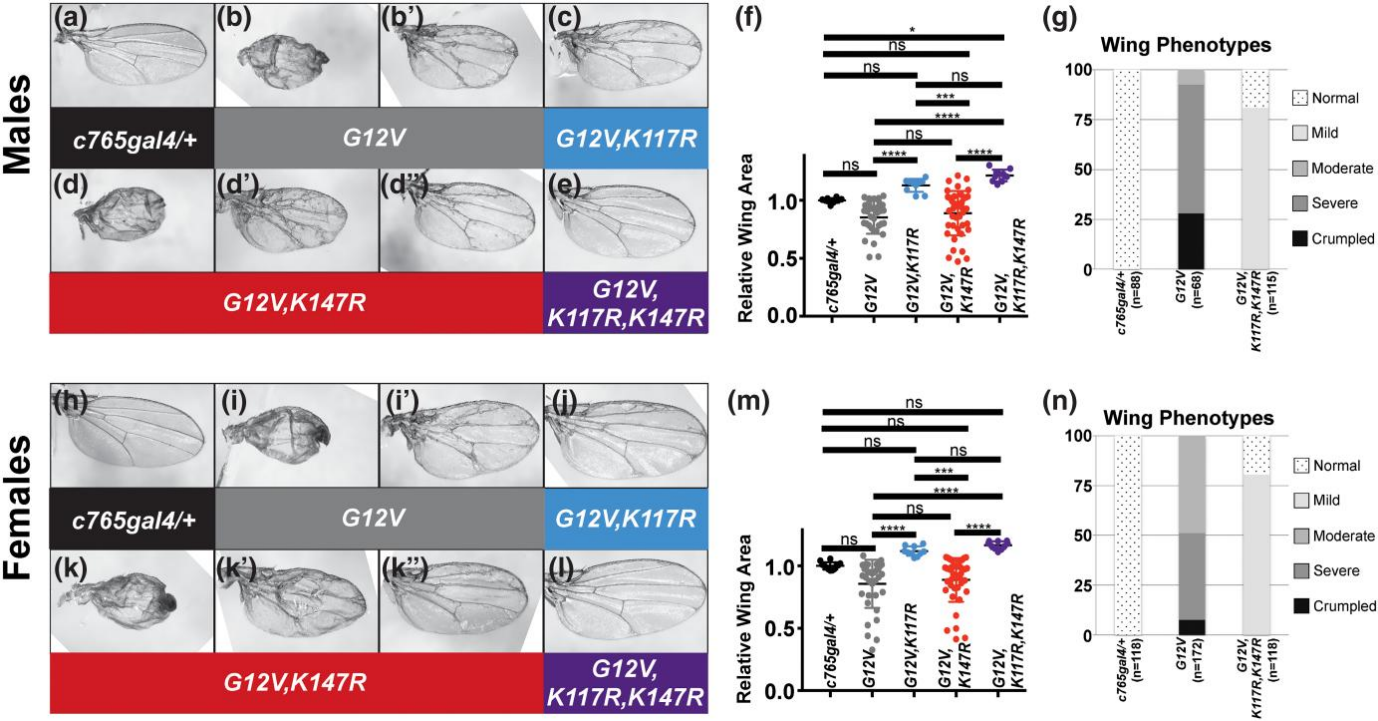
Mutations in lysines K117 and/or K147 suppress *Drosophila* Ras^G12V^ wing phenotypes at 21°C. Experiments were performed at 21°C; male wings are shown in a–g; female wings are shown in h–n. a, h) Control *c765-gal4/+* wing. b, bʹ, i, and iʹ) Expressing Ras^G12V^ in the wing using *c765-gal4* causes visible patterning phenotypes including differentiation of extra wing vein material. The degree of mispatterning varies from wings of reduced size from multiple abnormalities including ectopic wing veins, crumpling, and blisters (b, i) to normal to overgrown wings with ectopic wing veins (bʹ, iʹ). The range of sizes is evident when graphing wing size (shown later in f and m) or categorizing severity (shown later in g and n). c, j) Expressing Ras^G12V,K117R^ in the wing using *c765-gal4* results in significant suppression of the patterning abnormalities although some wing vein disruption is still obvious. Notably, there is little variability in wing size (shown later in f and m) in contrast to Ras^G12V^ wings. d, d′, dʺ, k, k′, and kʺ) Expressing Ras^G12V,K147R^ in the wing using *c765-gal4* results in a range of phenotypes such as wings resembling Ras^G12V^ wings including small wings with abnormalities (d, k) and normal to overgrown wings with abnormalities (dʹ, kʹ) but also a number of wings in which the patterning abnormalities are significantly suppressed (dʺ, kʺ). Wing sizes are variable (shown later in f and m). e, l) Expressing Ras^G12V,K117R,K147R^ in the wing using *c765-gal4* results in significant suppression of the patterning abnormalities. As with Ras^G12V,K117R^ wings, there is little variability in wing size (shown in f and m) in contrast to Ras^G12V^ wings. f, m) Wing sizes for wings in a–e and h–l were measured and graphed. g, n) Control wings and wings expressing Ras^G12V^ or Ras^G12V,K117R,K147R^ were scored in the categories of “normal” (no abnormalities—white, dotted in left bar and top portion of right bar), “mild” (some ectopic vein material, mostly where the longitudinal veins meet the wing margin—very light gray, bottom portion of right bar), “moderate” (moderate ectopic wings such as image in iʹ—medium gray, top portion of middle bar), “severe” (severe ectopic wing veins similar to the wing shown in dʹ—dark gray, middle portion of middle bar), or “crumpled” (crumpled and/or blistered wings such as the wings shown in b, d, i, and k—black, bottom portion of middle bar). Categorical scoring highlights that there is no overlap in phenotypes between Ras^G12V^ and Ras^G12V,K117R,K147R^ wings. For f and m, ns indicates not significant; *, **, ***, and **** indicate statistically significant; for *P* values, see Supplementary File 1. For statistical analysis of categorical scoring and *P* values for g and n, see Supplementary File 1. Genotypes in this figure are as follows: *w; c765-gal4/+* (a, h; left-most genotype in graphs in f, g, m, and n), *w; UAS Ras^G12V^/+; c765-gal4/+* (b, bʹ, i, and iʹ; second genotype in graphs in f, g, m, and n), *w; UAS Ras^G12V,K117R^/+; c765-gal4/+* (c, j; third genotype in graphs in f and m), *w; UAS Ras^G12V,K147R^/+; c765-gal4/+* (d, d′, dʺ, k, k′, and kʺ; fourth genotype in graphs in f and m), *w; UAS Ras^G12V,K117R,K147R^/+; c765-gal4/+* (e, l; right-most genotype in graphs in f, g, m, and n).

**Fig. 2.**
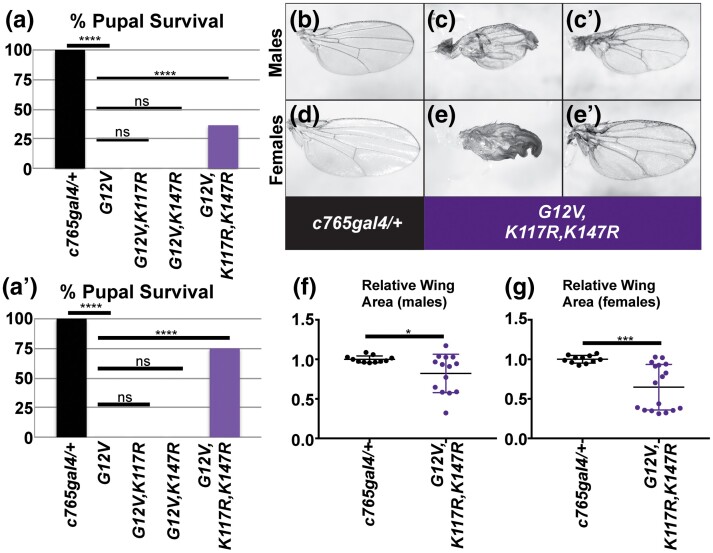
Concurrent mutation in K117 and K147 suppresses *Drosophila* Ras^G12V^ lethality at 25°C. Experiments were performed at 25°C; male wings are shown in b–cʹ and f; female wings are shown in d–eʹ and g). a, aʹ) The lethality of flies pupating on the vial walls was calculated by counting the number of empty pupal cases and dead pupae. In every trial, all or almost all *c765-gal4/+* flies survive to adulthood whereas *Ras^G12V^* showed 100% pupal lethality. We observed variability from trial to trial in terms of overall percent lethality for the *Ras^G12V,K117R^* (see Supplementary File 1) and *Ras^G12V,K117R,K147R^* genotypes but the trend was the same. a) Graph summarizing the percent pupal lethality for the indicated genotypes. Example of a trial with lower level survival of *Ras^G12V,K117R,K147R^* flies. ns indicates not significant by Fisher's exact test; **** indicates statistically significant in both chi-square and Fisher's exact tests; for *P* values, see Supplementary File 1. aʹ) Graph summarizing the percent pupal lethality for the indicated genotypes. Example of a trial with high survival of *Ras^G12V,K117R,K147R^* flies. ns indicates not significant by Fisher's exact test; **** indicates statistically significant in both chi-square and Fisher's exact tests; for *P* values, see Supplementary File 1. b, d) Control *c765-gal4/+* wing. c, cʹ, e, and eʹ) Expressing Ras^G12V,K117R,K147R^ in the wing using *c765-gal4* results in a range of phenotypes including wings resembling Ras^G12V^ wings from 21°C including small wings with abnormalities (c, e) but also a number of wings in which the patterning abnormalities are less obvious (cʹ, eʹ). Wing sizes are variable (shown in f and g). f, g) Wing sizes of wings from b–eʹ were measured and graphed in scatter plots. f) Graph summarizing the relative apparent male wing area from experiments shown in b–cʹ. g) Graph summarizing the relative apparent female wing area from experiments shown in d–eʹ. For data in f and g, ns indicates not significant; * and *** indicate statistically significant; for *P* values, see Supplementary File 1. Genotypes in this figure are: *w; c765-gal4/+* (b, d; left-most genotype in graphs in a, aʹ, f, and g), *w; UAS Ras^G12V^/+; c765-gal4/+* (second genotype in graphs in a and aʹ), *w; UAS Ras^G12V,K117R^/+; c765-gal4/+* (third genotype in graphs in a and aʹ), *w; UAS Ras^G12V,K147R^/+; c765-gal4/+* (fourth genotype in graphs in a and aʹ), *w; UAS Ras^G12V,K117R,K147R^/+; c765-gal4/+* (c, cʹ, e, and eʹ; right-most genotype in graphs in a, aʹ, f, and g).

**Fig. 3.**
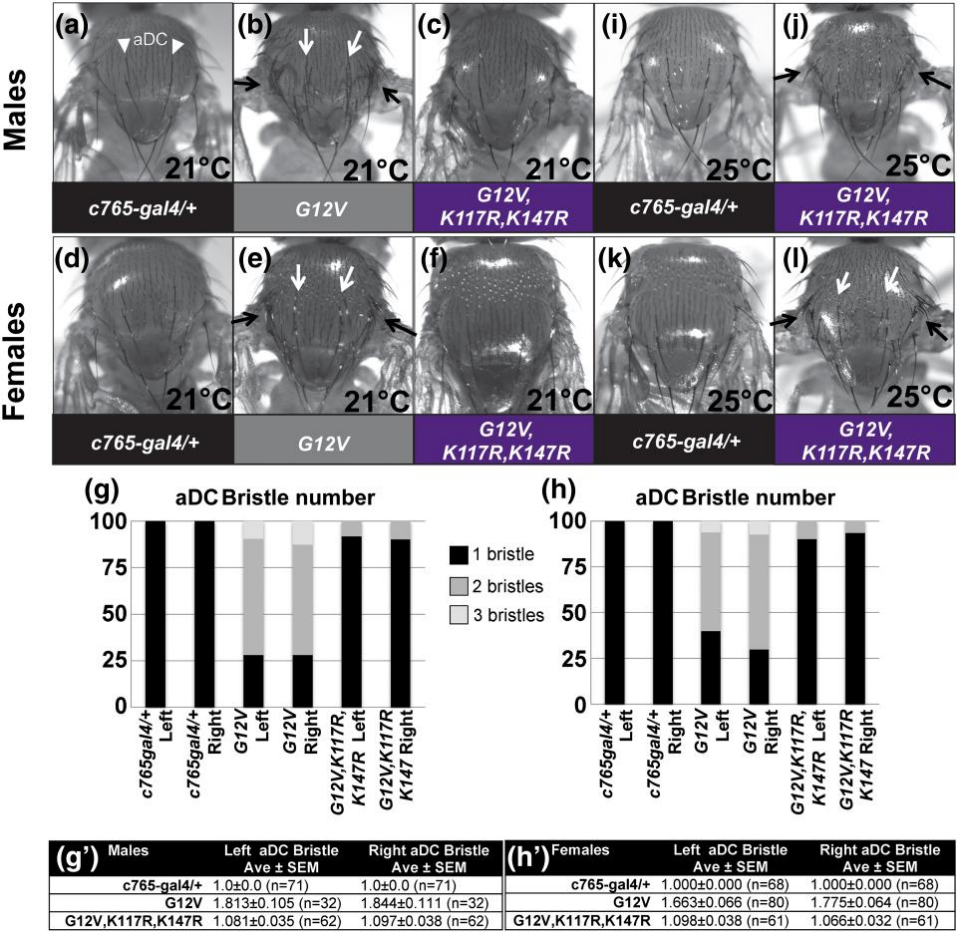
Concurrent mutation in K117 and K147 suppresses *Drosophila* Ras^G12V^ bristle phenotypes at 21°C. Experiments in a–f were performed at 21°C; experiments in i–l were performed at 25°C. a, d) Control *c765-gal4/+* thorax. aDC bristles are marked with white arrowheads in aʹ. b, and e) Expressing Ras^G12V^ using *c765-gal4* causes visible bristle phenotypes (arrows) on the thorax including extra bristles and disrupted bristle patterning. Extra bristles at the position of the aDC bristles are noted with white arrows. c, f) The bristle phenotypes are decreased in Ras^G12V,K117R,K147R^ flies. g, h) As shown in a, in wild-type flies, there are 2 aDC macrochaetes, 1 on each side. We scored the number of bristles at the aDC positions for each of these genotypes at 21°C. Flies expressing Ras^G12V^ showed 1–3 bristles at each aDC position. Graphs showing relative percent in each category are shown in g for males and in h for females. Categorical analysis of the categories of 1, 2, or 3 bristles at each aDC position and using chi-square or Fisher's exact tests indicated statistically significant difference between genotypes (see Supplementary File 1 for *P* values). This was suppressed in Ras^G12V,K117R,K147R^ flies. gʹ, hʹ) Tables summarizing the average number of bristles and SEM at each aDC position for each genotype for (gʹ) males and (hʹ) females. ANOVA analysis indicates a statistically significant increase in the average number of bristles for Ras^G12V^ compared to controls and statistically significant suppression in bristle number for Ras^G12V,K117R,K147R^ compared to Ras^G12V^ (see Supplementary File 1 for *P* values). i, k) Control *c765-gal4/+* thorax. j, l) Expressing Ras^G12V,K117R,K147R^ using *c76ap5-gal4* causes bristle phenotypes (arrows) on the thorax similar to Ras^G12V^ phenotypes at 21°C shown in b and c. Genotypes in this Figure are: *w; c765-gal4/+* (a, d, i, and k; left-most 2 bars in graphs in g and h), *w; UAS Ras^G12V^/+; c765-gal4/+* (b, e; middle 2 bars in graphs in g and h), *w; UAS Ras^G12V,K117R,K147R^/+; c765-gal4/+* (c, f, j, and l; right-most two bars in graphs in g and h).

Wings were measured using ImageJ software. Raw measurements in pixels and normalized measurements appearing in graphs are in Supplementary File 1. Wing size comparisons and categorical analysis were analyzed using GraphPad Prism software. Specifically, 1-way ANOVA analysis with multiple comparisons (Figs. 1f and m and 3g–hʹ) or unpaired *t*-tests (Fig. 2f and g) was used to assess statistical significance of changes in wing size. Chi-square analysis and Fisher's exact tests were applied as appropriate using contingency tables for categorical scoring of wing phenotypes (Fig. 1g and n), categorical analysis of pupal lethality (Fig. 2a and aʹ), and categorical analysis of anterior dorsocentral (aDC) bristle number (Fig. 3g and h). *P* values are listed in Supplementary File 1.

### Ras constructs and *Drosophila* transgenic lines

*UAS Ras^G12V,K117R^*, *UAS Ras^G12V,K147R^*, and *UAS Ras^G12V,K117R,K147R^* were cloned into pUAST-attB using the EcoRI and NotI restriction sites. FLAG and His6 sequences MDYKDDDDKRGSHHHHHHALE were added immediately after the EcoRI site (corresponding to the N-terminal nucleotide sequence GAATTCATGGATTACAAGGATGACGACGATAAGAGAGGATCGCATCACCATCACCATCACGCGCTCGAG, EcoRI site underlined) as we did previously with UAS Ras^G12V^ (Washington *et al.* 2020). An additional stop codon and a NotI site were added immediately after the original stop codon (corresponding to the sequence TAATAAGCGGCCGC, original stop codon underlined). The plasmids were sent to BestGene for injection and generation of transgenic lines at the attp40 locus. Lines were balanced with second chromosome balancers, and then each individual balanced line was allowed to homozygose. Homozygous lines were then maintained as true-breeding homozygous stocks. BestGene confirmed insertion at attp40, and we sequenced genomic DNA to confirm the sequence of each insert. Importantly, *UAS Ras^G12V,K117R^*, *UAS Ras^G12V,K147R^*, and *UAS Ras^G12V,K117R,K147R^* differ in sequence from *UAS Ras^G12V^* only at K117 and/or K147. These transgenes are listed in Supplementary Table 1, and protein sequences are listed in Supplementary Table 2.

### *Drosophila* experiments

Crosses were performed at the indicated temperatures on standard *Drosophila* medium as in our previous work (Yan *et al.* 2009; Yan *et al.* 2010; Washington *et al.* 2020). In each experimental trial, crosses were set up on food from the same batch, and all vials were kept in close proximity in the same box and experienced the same environment; if there were any environmental variables (such as variations in batches of food or slight fluctuations in temperature when incubator doors were opened), all vials in each trial experienced them simultaneously. We cannot rule out slight environmental fluctuations between trials or slight differences in batches of *Drosophila* food that may contribute to variations between trials.

### Image analysis and processing

Adult wings and other structures were photographed using a Nikon DS-Fi3 microscope camera and saved as TIFF files. Raw wing images were converted to grayscale using Adobe Photoshop. Brightness and contrast of wing images were adjusted using Adobe Photoshop to maximize clarity; adjustments were applied to the entire images. Genotypes are summarized in the figure legends, and identifiers are annotated in Supplementary Table 1.

### Pupal lethality

For experiments in Fig. 2a and aʹ, pupal cases were scored as empty (reflecting flies surviving to adulthood) or dead (in which dead pupae remained in the pupal case) and counted 18 days after egg-laying. Although activated Ras transgene expression can cause a delay beyond the 10-day developmental period, this is long enough to ensure all delayed pupae have eclosed. Moreover, dead pupae/pharate adults that have not eclosed are easily distinguished from live pupae given their black and shriveled appearance. Percent pupal lethality was then graphed in Fig. 2a and aʹ. Categorical analyses using chi-square and Fisher's exact test were applied as described in the statistical analysis section.

## Results and discussion

To address conservation of the role of K117 and K147 between mammalian Ras isoforms and *Drosophila* Ras, we utilized a Ras^G12V^ construct we created previously (Washington *et al.* 2020), similar versions of which have been widely used throughout the field for decades (Karim and Rubin 1998). The G12V substitution mutation biases Ras to the active GTP-loaded conformation, but Ras^G12V^ still undergoes a low rate of GTP hydrolysis and nucleotide exchange (Hunter *et al.* 2015). This makes Ras^G12V^ an excellent background in which to interrogate the reported mammalian roles of K117 and K147 ubiquitination in Ras activation through phenotype severity. We created arginine substitution mutations (to preserve positive charge but prevent conjugation to ubiquitin) in the G12V background to assess the contribution of K117 and K147 to Ras activation: double mutants Ras^G12V, K117R^ and Ras^G12V,K147R^ and triple mutant Ras^G12V,K117R,K147R^. All transgenes carry the same tags and were inserted at the same attp40 location in the genome to rule out position insertion effects. If neither K117 nor K147 contributes to Ras activation, these double and triple mutants will phenocopy Ras^G12V^. If only 1 lysine contributes to Ras activation, then that specific double mutant should show phenotypic suppression compared to a Ras^G12V^ control and will phenocopy the suppression seen in the triple mutant. If both K117 and K147 contribute to Ras activation, then the triple mutant should show suppression compared to a Ras^G12V^ control and greater suppression than that shown by each individual double mutant.

To assess Ras overexpression phenotypes in the wing, we utilized gal4 driver *c765-gal4*, which drives expression generally across the wing (Guillen *et al.* 1995; de Celis *et al.* 1996) and is typically used as a pan-wing driver but also drives expression in other tissues including generalized expression in the thorax (Gomez-Skarmeta *et al.* 1996; Yang *et al.* 2012), in leg discs (Azpiazu and Morata 2002), and in the brain (Rodan *et al.* 2002). Driving Ras^G12V^ in the wing using *c765-gal4* at 21°C results in differentiation of extra and ectopic wing vein material and thickened veins (Fig. 1b, bʹ, i, and iʹ). Ras can promote proliferation and overgrowth, and some wings reach normal size or increase in size (Fig. 1bʹ and iʹ); however, the differentiation of extra wing veins can happen at the expense of wing material thus reducing overall wing size (Fig. 1b and i) compared to a control wing (Fig. 1a and h). The variety of wing sizes is reflected in scatter plots in Fig. 1f and m, and categorical scoring of variable wing phenotypes is shown in Fig. 1g and n. In addition, these wings can have other abnormalities including blisters and folds that cause the wing to crumple resulting in a reduction in apparent wing size. Driving Ras^G12V,K117R^ in the wing with *c765-gal4* at 21°C resulted in obvious suppression of the variable wing size and wing differentiation phenotypes including a statistically significant difference in wing size (Fig. 1c and j and scatter plots in Fig. 1f and m; Supplementary File 1) compared to Ras^G12V^ wings (Fig. 1b, bʹ, i, and iʹ; Supplementary File 1). There was a reproducible trend of increased wing size compared to control wings (Fig. 1f and m), but this was typically not statistically significant (Supplementary File 1). The suppression of wing vein phenotypes might have allowed a Ras-induced overgrowth phenotype to be observed more frequently resulting in this trend; alternatively, this lysine might be involved in distinguishing between downstream biological outputs of wing patterning and growth. Driving Ras^G12V,K147R^ with *c765-gal4* at 21°C resulted in a highly variable phenotype ranging from wings similar to Ras^G12V^ wings (Fig. 1d, dʹ, k, and kʹ; Supplementary File 1) to individual wings showing some suppression of both the wing vein phenotypes and the reduced wing size phenotypes (Fig. 1dʺ and kʺ), but there was no statistically significant difference in wing size in the population of Ras^G12V,K147R^ wings compared to Ras^G12V^ wings (scatter plot shown in Fig. 1f and m). The variable phenotype of Ras^G12V,K147R^ is difficult to interpret in comparison to Ras^G12V^. Therefore, we also utilized triple mutant Ras^G12V,K117R,K147R^. Driving Ras^G12V,K117R,K147R^ with *c765-gal4* at 21°C led to more dramatic suppression of the wing vein phenotypes than either double mutant (Fig. 1e and l) and suppression of the variable size phenotypes (Fig. 1f and m) including statistically significantly different wing size compared to Ras^G12V,K147R^ double mutant wings and Ras^G12V^ wings (Supplementary File 1). Categorical scoring of phenotypic severity in Ras^G12V,K117R,K147R^ wings highlights this suppression compared to Ras^G12V^ wings (Fig. 1g and n; Supplementary File 1). As with the Ras^G12V,K117R^ double mutant, there was a reproducible trend of increased wing size compared to control wings (Fig. 1f and m). In some trials, this was statistically significant (example shown in Fig. 1f for males; Supplementary File 1) but not always (example shown in Fig. 1m for females; Supplementary File 1). The increased suppression of the wing vein phenotypes in the triple mutant Ras^G12V,K117R,K147R^ compared to the Ras^G12V,K117R^ double mutant is consistent with the K147R mutation contributing to the suppression.

Driving Ras^G12V^ with *c765-gal4* at 25°C results in lethality. Driving Ras^G12V,K117R^ with *c765-gal4* at 25°C led to some escapers surviving to adulthood in some trials (Supplementary File 1). In most cases, this was not statistically significant survival compared to Ras^G12V^ controls. The majority of trials resulted in no survival to adulthood of these flies (Fig. 2a and aʹ). Driving Ras^G12V,K147R^ with *c765-gal4* at 25°C resulted in lethality as with Ras^G12V^ (Fig. 2a and aʹ). Driving triple mutant Ras^G12V,K117R,K147R^ with *c765-gal4* at 25°C resulted in dramatic and statistically significant suppression of lethality compared to Ras^G12V^ controls (Supplementary File 1). We saw variability in the extent of survival but reproducibly observed at least a third of pupae survived to adulthood (Fig. 2a) in some trials, whereas in others, more than 75% of pupae surviving to adulthood (Fig. 2aʹ). Most Ras^G12V,K117R,K147R^-expressing wings showed clear Ras gain-of-function phenotypes (Fig. 2c, cʹ, e, and eʹ; scatter plot shown in Fig. 2f and g) compared to control flies (Fig. 2b and d; Supplementary File 1) and consistent with Ras^G12V^ phenotypes from 21°C (Fig. 1). Because the Gal4/UAS system is temperature responsive, having to increase the temperature to 25° to elicit a phenotype in Ras^G12V,K117R,K147R^ similar to Ras^G12V^ at 21°C further supports the differences in severities between these mutants. In addition, the dramatic suppression of lethality for Ras^G12V,K117R,K147R^ despite no significant suppression of lethality for Ras^G12V,K117R^ and Ras^G12V,K147R^ suggests that both K117R and K147R mutations contribute to the suppression of lethality.

While *c765-gal4* is often used for its ability to drive expression in the wing, it is important to note that this driver directs expression in other tissues including generally in the thorax (Gomez-Skarmeta *et al.* 1996; Yang *et al.* 2012), in the leg discs (Azpiazu and Morata 2002), in the mushroom body, in the fan-shaped body, in the ellipsoid body of the adult brain (Rodan *et al.* 2002), and possibly in other tissues. Lethality might result from broader expression than in the wing. In fact, even at 21°C, we see bristle phenotypes on the thorax of Ras^G12V^-expressing flies (Fig. 3b and e) compared to control flies (Fig. 3a and d). These phenotypes are somewhat suppressed in triple mutant Ras^G12V,K117R,K147R^ (Fig. 3c and f). To highlight this suppression, we counted the number of bristles at the position of the aDC bristles. Control flies (Fig. 3a and d; Supplementary File 1) typically have 1 aDC bristle on the left and 1 on the right. In Ras^G12V^-expressing flies, we counted 1–3 bristles at this position (although we have not addressed if this was due to bristle duplication or ectopic bristles in the region). We performed categorical analysis (Fig. 3g and h; Supplementary File 1) and ANOVA analysis (Fig. 3gʹ and hʹ; Supplementary File 1) both of which showed statistically significant differences in Ras^G12V^ compared to controls and statistically significant suppression in Ras^G12V,K117R,K147R^ compared to Ras^G12V^. Interestingly, those Ras^G12V,K117R,K147R^-expressing flies that survived to adulthood at 25°C exhibited similar bristle phenotypes (Fig. 3j and l) to Ras^G12V^ flies from 21°C (Fig. 3b and e) not seen in control flies (Fig. 3i and k). These bristle phenotypes are consistent with *c765-gal4*-induced expression in the thorax reported previously (Gomez-Skarmeta *et al.* 1996; Yang *et al.* 2012) and Ras^G12V^ and bristle phenotypes on the thorax reported for *apterous-gal4* (*ap-gal4*) (Culí *et al.* 2001), which expresses in the pattern of the *apterous* gene including in the third instar dorsal wing, tarsal segment 4, brain, thorax, and elsewhere (Guarner *et al.* 2014; Kim *et al.* 2004; Cohen *et al.* 1992; Moreno and Morata 1999; Azpiazu and Morata 2002; Paul *et al.* 2013).

As noted earlier, if both K117 and K147 contribute to Ras activation, then triple mutant Ras^G12V,K117R,K147R^ would show suppressed phenotypes compared to Ras^G12V^ and greater suppression than either Ras^G12V,K117R^ or Ras^G12V,K147R^ alone. Taken together, the different degrees of suppression of Ras^G12V^ phenotypes in Figs. 1 and 2 by Ras^G12V,K117R^ and Ras^G12V,K117R,K147R^ are consistent with both lysines K117 and K147 playing a role in the status of Ras activation in *Drosophila* as seen in mammals. Arginine substitution preserves the positive charge of lysine while preventing modifications such as ubiquitination and acetylation. It is formally possible these substitution mutants are inactivating by affecting Ras structure or its ability to adopt an active conformation, and we have not ruled out such structural defects from K117R and K147R mutants in *Drosophila* Ras. However, work in mammalian systems showed that K147R in mammalian KRas maintained an active conformation capable of binding the RBD (Sasaki *et al.* 2011). In addition, K117R mutations are found in HRas in the rasopathy Costello syndrome and in KRas in colorectal cancer (Kerr *et al.* 2006; Haigis 2017), thus exhibiting gain-of-function phenotypes. Moreover, although triple mutant Ras^G12V,K117R,K147R^ showed suppressed phenotypes compared to Ras^G12V^, it exhibited clear Ras gain-of-function wing (Figs. 1 and 2) and bristle (Fig. 3) phenotypes at 25°C confirming that it can adopt an active Ras configuration.

An alternate explanation for the activating role of K117 and K147 is modification at these sites. K117 is a site of activating ubiquitination in mammals, and K147 undergoes both activating ubiquitination and activating acetylation events (Akimov *et al.* 2018; Filipčík *et al.* 2017; Wagner *et al.* 2012; Sasaki *et al.* 2011; Baker *et al.* 2013; Udeshi *et al.* 2013; Yoshino *et al.* 2019; Mertins *et al.* 2013; Knyphausen *et al.* 2016; Song *et al.* 2016). Our data are consistent with a highly conserved role for K117 and K147 modification in activating Ras; we speculate that modification of these lysines was a mechanism for controlling Ras activity in a common ancestor between flies and mammals. The allele phenotypes in this work are consistent with but do not distinguish between activating ubiquitination and acetylation. Future work will be required to explore the modifications at each lysine to elucidate the specific biological roles of ubiquitination and/or acetylation at these sites and to further understand the activating nature of K117R single mutations vs the loss of function we observe here for K117R mutation in the G12V background. We speculate that K117 ubiquitination has a much greater effect on activity than K117 arginine substitution. The E3 ubiquitin ligases that place the ubiquitin at K117 and K147 would play important roles in Ras signaling dynamics; the Ras^G12V,K117R^ and Ras^G12V,K147R^ transgenes would be useful tools in future work to identify and evaluate these E3 enzymes and explore their role in development and disease.

## Supplementary Material

jkad201_Supplementary_Data

## Data Availability

*Drosophila* strains used in this work (listed in Supplementary Table 1, protein sequences listed in Supplementary Table 2; cloning sites and nucleotide sequence of N- and C-termini listed in Materials and methods section) are available upon request. Raw data, normalized data for graphs in Figs. 1–3, and *P* values are listed in Supplementary File 1. The authors affirm that all data necessary for interpreting the data and drawing conclusions are present within the article text, the figures, tables, and Supplementary File 1. Supplemental material available at G3 online.

## References

[jkad201-B1] Akimov V , Barrio-Hernandez I, Hansen SVF, Hallenborg P, Pedersen AK, Bekker-Jensen DB, Puglia M, Christensen SDK, Vanselow JT, Nielsen MM, et al 2018. Ubisite approach for comprehensive mapping of lysine and N-terminal ubiquitination sites. Nat Struct Mol Biol. 25(7):631–640. doi:10.1038/s41594-018-0084-y. 29967540

[jkad201-B2] Azpiazu N , Morata G. 2002. Distinct functions of homothorax in leg development in Drosophila. Mech Dev. 119(1):55–67. doi:10.1016/S0925-4773(02)00295-2. 12385754

[jkad201-B3] Baker R , Wilkerson EM, Sumita K, Isom DG, Sasaki AT, Dohlman HG, Campbell SL. 2013. Differences in the regulation of K-Ras and H-Ras isoforms by monoubiquitination. J Biol Chem. 288(52):36856–36862. doi:10.1074/jbc.C113.525691. 24247240 PMC3873545

[jkad201-B4] Bigenzahn JW , Collu GM, Kartnig F, Pieraks M, Vladimer GI, Heinz LX, Sedlyarov V, Schischlik F, Fauster A, Rebsamen M, et al 2018. LZTR1 is a regulator of RAS ubiquitination and signaling. Science. 362(6419):1171–1177. doi:10.1126/science.aap8210. 30442766 PMC6794158

[jkad201-B5] Cohen B , McGuffin ME, Pfeifle C, Segal D, Cohen SM. 1992. Apterous, a gene required for imaginal disc development in *Drosophila* encodes a member of the LIM family of developmental regulatory proteins. Genes Dev. 6(5):715–729. doi:10.1101/gad.6.5.715. 1349545

[jkad201-B6] Culí J , Martín-Blanco E, Modolell J. 2001. The EGF receptor and N signalling pathways act antagonistically in *Drosophila* mesothorax bristle patterning. Development. 128(2):299–308. doi:10.1242/dev.128.2.299. 11124124

[jkad201-B7] de Celis JF , Barrio R, Kafatos FC. 1996. A gene complex acting downstream of *dpp* in *Drosophila* wing morphogenesis. Nature. 381(6581):421–424. doi:10.1038/381421a0. 8632798

[jkad201-B8] Filipčík P , Curry JR, Mace PD. 2017. When worlds collide-mechanisms at the interface between phosphorylation and ubiquitination. J Mol Biol. 429(8):1097–1113. doi:10.1016/j.jmb.2017.02.011. 28235544

[jkad201-B9] Gomez-Skarmeta JL , Diez del Corral R, de la Calle-Mustienes E, Ferré-Marcó D, Modolell J. 1996. Araucan and caupolican, two members of the novel iroquois complex, encode homeoproteins that control proneural and vein-forming genes. Cell. 85(1):95–105. doi:10.1016/S0092-8674(00)81085-5. 8620542

[jkad201-B10] Guarner A , Manjón C, Edwards K, Steller H, Suzanne M, Sánchez-Herrero E. 2014. The zinc finger homeodomain-2 gene of *Drosophila* controls Notch targets and regulates apoptosis in the tarsal segments. Dev Biol. 385(2):350–365. doi:10.1016/j.ydbio.2013.10.011. 24144920

[jkad201-B11] Guillen I , Mullor JL, Capdevila J, Sanchez-Herrero E, Morata G, Guerrero I. 1995. The function of engrailed and the specification of *Drosophila* wing pattern. Development. 121(10):3447–3456. doi:10.1242/dev.121.10.3447. 7588077

[jkad201-B12] Haigis K . 2017. KRAS alleles: the devil is in the detail. Trends Cancer. 3(10):686–697. doi:10.1016/j.trecan.2017.08.006. 28958387 PMC5824632

[jkad201-B13] Hobbs GA , Gunawardena HP, Baker R, Campbell SL. 2013. Site-specific monoubiquitination activates Ras by impeding GTPase-activating protein function. Small GTPases. 4(3):186–192. doi:10.4161/sgtp.26270. 24030601 PMC3976977

[jkad201-B14] Hunter JC , Manandhar A, Carrasco MA, Gurbani D, Gondi S, Westover KD. 2015. Biochemical and structural analysis of common cancer-associated KRAS mutations. Mol Cancer Res. 13(9):1325–1335. doi:10.1158/1541-7786.MCR-15-0203. 26037647

[jkad201-B15] Jura N , Scotto-Lavino E, Sobczyk A, Bar-Sagi D. 2006. Differential modification of Ras proteins by ubiquitination. Mol Cell. 21(5):679–687. doi:10.1016/j.molcel.2006.02.011. 16507365

[jkad201-B16] Karim FD , Rubin GM. 1998. Ectopic expression of activated Ras1 induces hyperplastic growth and increased cell death in *Drosophila* imaginal tissues. Development. 125(1):1–9. doi:10.1242/dev.125.1.1. 9389658

[jkad201-B17] Kerr B , Delrue MA, Sigaudy S, Perveen R, Marche M, Burgelin I, Stef M, Tang B, Eden OB, O’Sullivan J , et al Genotype-phenotype correlation in Costello syndrome: HRAS mutation analysis in 43 cases. J Med Genet 2006; 43(5): 401–405. doi:10.1136/jmg.2005.040352. 16443854 PMC2564514

[jkad201-B18] Kim M , Cha GH, Kim S, Lee JH, Park J, Koh H, Choi KY, Chung J. 2004. MKP-3 has essential roles as a negative regulator of the Ras/mitogen-activated protein kinase pathway during *Drosophila* development. Mol Cell Biol. 24(2):573–583. doi:10.1128/MCB.24.2.573-583.2004. 14701731 PMC343793

[jkad201-B19] Knyphausen P , Lang F, Baldus L, Extra A, Lammers M. 2016. Insights into K-Ras 4B regulation by post-translational lysine acetylation. Biol Chem. 397(10):1071–1085. doi:10.1515/hsz-2016-0118. 27176741

[jkad201-B20] Mertins P , Qiao JW, Patel J, Udeshi ND, Clauser KR, Mani DR, Burgess MW, Gillette MA, Jaffe JD, Carr SA. 2013. Integrated proteomic analysis of post-translational modifications by serial enrichment. Nat Methods. 10(7):634–637. doi:10.1038/nmeth.2518. 23749302 PMC3943163

[jkad201-B21] Moreno E , Morata G. 1999. Caudal is the Hox gene that specifies the most posterior *Drosophile* segment. Nature. 400(6747):873–877. doi:10.1038/23709. 10476966

[jkad201-B22] Paul L , Wang SH, Manivannan SN, Bonanno L, Lewis S, Austin CL, Simcox A. 2013. Dpp-induced Egfr signaling triggers postembryonic wing development in *Drosophila*. Proc Natl Acad Sci U S A. 110(13):5058–5063. doi:10.1073/pnas.1217538110. 23479629 PMC3612653

[jkad201-B23] Rodan AR , Kiger JA, Heberlein U. 2002. Functional dissection of neuroanatomical loci regulating ethanol sensitivity in *Drosophila*. J Neurosci. 22(21):9490–9501. doi:10.1523/JNEUROSCI.22-21-09490.2002. 12417673 PMC6758036

[jkad201-B24] Sasaki AT , Carracedo A, Locasale JW, Anastasiou D, Takeuchi K, Kahoud ER, Haviv S, Asara JM, Pandolfi PP, Cantley LC. 2011. Ubiquitination of K-Ras enhances activation and facilitates binding to select downstream effectors. Sci Signal. 4(163):ra13. doi:10.1126/scisignal.2001518. 21386094 PMC3437993

[jkad201-B25] Song HY , Biancucci M, Kang HJ, O’Callaghan C, Park SH, Principe DR, Jiang H, Yan Y, Satchell KF, Raparia K, et al 2016. SIRT2 deletion enhances KRAS-induced tumorigenesis in vivo by regulating K147 acetylation status. Oncotarget. 7(49):80336–80349. doi:10.18632/oncotarget.12015. 27637077 PMC5340253

[jkad201-B26] Steklov M , Pandolfi S, Baietti MF, Batiuk A, Carai P, Najm P, Zhang M, Jang H, Renzi F, Cai Y, et al 2018. Mutations in LZTR1 drive human disease by dysregulating RAS ubiquitination. Science. 362(6419):1177–1182. doi:10.1126/science.aap7607. 30442762 PMC8058620

[jkad201-B27] Udeshi ND , Svinkina T, Mertins P, Kuhn E, Mani DR, Qiao JW, Carr SA. 2013. Refined preparation and use of anti-diglycine remnant (K-ε-GG) antibody enables routine quantification of 10,000s of ubiquitination sites in single proteomics experiments. Mol Cell Proteomics. 12(3):825–831. doi:10.1074/mcp.O112.027094. 23266961 PMC3591673

[jkad201-B28] Wagner SA , Beli P, Weinert BT, Schölz C, Kelstrup CD, Young C, Nielsen ML, Olsen JV, Brakebusch C, Choudhary C. 2012. Proteomic analyses reveal divergent ubiquitylation site patterns in murine tissues. Mol Cell Proteomics. 11(12):1578–1585. doi:10.1074/mcp.M112.017905. 22790023 PMC3518112

[jkad201-B29] Washington C , Chernet R, Gokhale RH, Martino-Cortez Y, Liu H-Y, Rosenberg AM, Shahar S, Pfleger CM. 2020. A conserved, N-terminal tyrosine signal directs Ras for inhibition by Rabex-5. PLoS Genet. 16(6):e1008715. doi:10.1371/journal.pgen.1008715. PMC7329146 32559233

[jkad201-B30] Xu L , Lubkov V, Taylor LJ, Bar-Sagi D. 2010. Feedback regulation of Ras signaling by Rabex-5-mediated ubiquitination. Curr Biol. 20(15):1372–1377. doi:10.1016/j.cub.2010.06.051. 20655225 PMC3436604

[jkad201-B31] Yan H , Chin ML, Horvath EA, Kane EA, Pfleger CM. 2009. Impairment of ubiquitylation by mutation in *Drosophila* E1 promotes both cell-autonomous and non-cell-autonomous Ras-ERK activation in vivo. J Cell Sci. 122(9):1461–1470. doi:10.1242/jcs.042267. 19366732 PMC2721006

[jkad201-B32] Yan H , Jahanshahi M, Horvath EA, Liu HY, Pfleger CM. 2010. Rabex-5 ubiquitin ligase activity restricts Ras signaling to establish pathway homeostasis in vivo in *Drosophila*. Curr Biol. 20(15):1378–1382. doi:10.1016/j.cub.2010.06.058. 20655224 PMC2938185

[jkad201-B33] Yang M , Hatton-Ellis E, Simpson P. 2012. The kinase Sgg modulates temporal development of macrochaetes in *Drosophila* by phosphorylation of Scute and Pannier. Development. 139(2):325–334. doi:10.1242/dev.074260. 22159580 PMC3243096

[jkad201-B34] Yoshino H , Yin G, Kawaguchi R, Popov KI, Temple B, Sasaki M, Kofuji S, Wolfe K, Kofuji K, Okumura K, et al 2019. Identification of lysine methylation in the core GTPase domain by GoMADScan. PLoS One. 14(8):e0219436. doi:10.1371/journal.pone.0219436. PMC6685615 31390367

