# Supplementary material for: Lysines K117 and K147 play conserved roles in Ras activation from Drosophila to mammals
Source: G3 (Bethesda). 2023 Sep 4;13(11):jkad201. doi: 10.1093/g3journal/jkad201 (PMC10627255; doi:10.1093/g3journal/jkad201)
Supplement: jkad201_Supplementary_Data [file jkad201_supplementary_data.zip › Supplemental_Table_S1_G3-2023-404401.docx]

**Supplemental Table 1: Table of reagents used with corresponding identifiers.**

| **Drosophila Strains** | | |
| --- | --- | --- |
| **Strain** | **Source** | **Identifier** |
| *w^1118^* | The fly community and Bloomington Drosophila Stock Center (BDSC) | BL-3605, BL-5905 and others  RRID:BDSC_3605, RRID:BDSC_5905 |
| *w; c765-gal4* | BDSC |  |
| *UAS Flag-His6-Ras^G12V^* | Washington et al., 2020 |  |
| *UAS Flag-His6-Ras^G12V,K117R^* | This study |  |
| *UAS Flag-His6-Ras^G12V,K147R^* | This study |  |
| *UAS Flag-His6-Ras^G12V,K117R,K147R^* | This study |  |
| **Software** | | |
| Image J |  | https://imagej.nih.gov/ij/ |
| Adobe Photoshop |  | https://www.adobe.com/products/photoshop.html |
| GraphPad Prism |  | https://www.graphpad.com/scientific-software/prism/ |
| Microsoft Excel |  | https://www.microsoft.com/Microsoft/Excel/ |
