# Supplementary material for: Lysines K117 and K147 play conserved roles in Ras activation from Drosophila to mammals
Source: G3 (Bethesda). 2023 Sep 4;13(11):jkad201. doi: 10.1093/g3journal/jkad201 (PMC10627255; doi:10.1093/g3journal/jkad201)
Supplement: jkad201_Supplementary_Data [file jkad201_supplementary_data.zip › Supplemental_Table_S2_G3-2023-404401.docx]

**Supplemental Table 2: Table of protein sequences for Ras constructs.**

| Construct (as listed in Table 1) | Protein sequence  Mutations: **bold, red**  Tags:  FLAG=**DYKDDDDK**, yellow  His6=**HHHHHH**, green |
| --- | --- |
| UAS Flag-His6-Ras^G12V^ (from Washington et al., 2020) | M**DYKDDDDK**RGS**HHHHHH**ALEMTEYKLVVVGA**V**GVGKSALTIQLIQNHFVDEYDPTIEDSYRKQVVIDGETCLLDILDTAGQEEYSAMRDQYMRTGEGFLLVFAVNSAKSFEDIGTYREQIKRVKDAEEVPMVLVGNKCDLASWNVNNEQAREVAKQYGIPYIETSAKTRMGVDDAFYTLVREIRKDKDNKGRRGRKMNKPNRRFKCKML |
| UAS Flag-His6-Ras^G12V,K117R^ | M**DYKDDDDK**RGS**HHHHHH**ALEMTEYKLVVVGA**V**GVGKSALTIQLIQNHFVDEYDPTIEDSYRKQVVIDGETCLLDILDTAGQEEYSAMRDQYMRTGEGFLLVFAVNSAKSFEDIGTYREQIKRVKDAEEVPMVLVGN**R**CDLASWNVNNEQAREVAKQYGIPYIETSAKTRMGVDDAFYTLVREIRKDKDNKGRRGRKMNKPNRRFKCKML |
| UAS Flag-His6-Ras^G12V,K147R^ | M**DYKDDDDK**RGS**HHHHHH**ALEMTEYKLVVVGA**V**GVGKSALTIQLIQNHFVDEYDPTIEDSYRKQVVIDGETCLLDILDTAGQEEYSAMRDQYMRTGEGFLLVFAVNSAKSFEDIGTYREQIKRVKDAEEVPMVLVGNKCDLASWNVNNEQAREVAKQYGIPYIETSA**R**TRMGVDDAFYTLVREIRKDKDNKGRRGRKMNKPNRRFKCKML |
| UAS Flag-His6-Ras^G12V,K117R,K147R^ | M**DYKDDDDK**RGS**HHHHHH**ALEMTEYKLVVVGA**V**GVGKSALTIQLIQNHFVDEYDPTIEDSYRKQVVIDGETCLLDILDTAGQEEYSAMRDQYMRTGEGFLLVFAVNSAKSFEDIGTYREQIKRVKDAEEVPMVLVGN**R**CDLASWNVNNEQAREVAKQYGIPYIETSA**R**TRMGVDDAFYTLVREIRKDKDNKGRRGRKMNKPNRRFKCKML |
